# Nanotopographic Substrates of Poly (Methyl Methacrylate) Do Not Strongly Influence the Osteogenic Phenotype of Mesenchymal Stem Cells In Vitro

**DOI:** 10.1371/journal.pone.0090719

**Published:** 2014-03-03

**Authors:** Isaac A. Janson, Yen P. Kong, Andrew J. Putnam

**Affiliations:** 1 Department of Material Science and Engineering, University of Michigan, Ann Arbor, Michigan, United States of America; 2 Department of Biomedical Engineering, University of Michigan, Ann Arbor, Michigan, United States of America; University of California at Davis, United States of America

## Abstract

The chemical, mechanical, and topographical features of the extracellular matrix (ECM) have all been documented to influence cell adhesion, gene expression, migration, proliferation, and differentiation. Topography plays a key role in the architecture and functionality of various tissues *in vivo*, thus raising the possibility that topographic cues can be instructive when incorporated into biomaterials for regenerative applications. In the literature, there are discrepancies regarding the potential roles of nanotopography to enhance the osteogenic phenotype of mesenchymal stem cells (MSC). In this study, we used thin film substrates of poly(methyl methacrylate) (PMMA) with nanoscale gratings to investigate the influence of nanotopography on the osteogenic phenotype of MSCs, focusing in particular on their ability to produce mineral similar to native bone. Topography influenced focal adhesion size and MSC alignment, and enhanced MSC proliferation after 14 days of culture. However, the osteogenic phenotype was minimally influenced by surface topography. Specifically, alkaline phosphatase (ALP) expression was not increased on nanotopographic films, nor was calcium deposition improved after 21 days in culture. Ca: P ratios were similar to native mouse bone on films with gratings of 415 nm width and 200 nm depth (G415) and 303 nm width and 190 nm depth (G303). Notably, all surfaces had Ca∶P ratios significantly lower than G415 films. Collectively, these data suggest that, PMMA films with nanogratings are poor drivers of an osteogenic phenotype.

## Introduction

Due to an aging population and the continued prevalence of bone defects worldwide, orthopedic procedures are increasingly needed each year [1], [2]. Revision surgeries continue to rise as well [1]. The cost of orthopedic injuries is estimated to be $17–20 billion annually in the United States [3]. Thus, strategies which enhance knowledge of bone formation or enhance current clinical practices are desirable. Improvements may prolong implant lifetime, reduce the need for revisions, and drive down the economic impact.

Mesenchymal stem cells (MSCs) are self-renewing marrow derived cells that are multipotent [4]. Because of the relative ease of isolation and high degree of plasticity, MSCs have been explored for tissue engineering and regenerative medicine applications [5]–[8]. MSCs' plasticity includes the ability to differentiate into chondrogenic, adipogenic and osteogenic phenotypes. Differentiation along an osteogenic lineage may be useful for bone tissue engineering strategies when combined with scaffolds to produce functional bone for various orthopedic therapies [2].

A cell's environment is critical to its function and behavior, and an important feature of the cellular microenvironment is the extracellular matrix (ECM). Cells sense and respond to both chemical and physical cues within the ECM, including adhesive ligands, mechanical properties, and topography [8], [9]. Cells adhere to the ECM's various adhesion motifs through transmembrane integrin receptors, which are capable of transducing adhesive signals into biochemical signals and which physically connect the ECM to the cell's underlying cytoskeleton. Different ECM ligands may engage different integrin receptors, and specific integrins are known to regulate osteogenic differentiation [10]. Matrix mechanical properties have also been shown to influence osteogenic differentiation, with more rigid substrates driving osteogenic differentiation in 2D cell cultures [11]–[13]. Other studies have shown micro- and nanotopography of varied surface chemistries to influence osteogenic differentiation as well [2]–[4], [10], [14]–[20].

Bone is a hierarchical tissue that is mainly composed of type I collagen, hydroxyapatite (HA), and water. Human bone structure ranges many orders of magnitude in size: from whole bones nearly one meter in length to the individual collagen triple helices that are approximately 300 nm long and have a diameter of 1.5 nm [21]. Collagen is essential for HA formation [22]; together HA and collagen form a highly aligned composite matrix that gives bone its toughness and strength [21], [23]. Thus, attempts to reproduce *in vivo* mineral *in vitro* may be enhanced by mimicking the structure of native bone. Many studies have reported on the influence of micro- and nano-topographies on the proliferation, genotype, and protein levels of osteoprogenitor cells, but rarely have they quantitatively assessed the deposited mineral. Hence, it is unclear whether topography drives a phenotype capable of producing mineral *in vitro* or *in vivo* similar to physiologically relevant bone. Ultimately, control of osteogenic differentiation via topography may be desirable for bone and orthopedic implant applications if indeed mineral production can be enhanced or if the mineral produced is similar to native bone.

In this study, we created an approximate replica of the nanotopographic structure of bone using an idealized surface of poly(methyl methacrylate) (PMMA) to investigate the role of surface nanotopography in driving the osteogenic differentiation of MSCs. Clinically, PMMA is used as a bone cement in orthopedic applications [24], [25]. Thus, motivated by the potential to enhance osteointegration and bone healing via imprinted nanotopographic cues on an FDA-approved orthopedic material, we focused specifically on mineralization as a functional metric of the mature bone phenotype. We hypothesized that our nanoPMMA surfaces with aligned features on the order of collagen fibrils would enhance mineral quantity. To test this hypothesis, we used PMMA films manufactured via capillary assisted ultra-violet (UV) lithography and characterized with atomic force microscopy (AFM) and scanning electron microscopy (SEM) to validate their submicron dimensions. MSCs were subsequently seeded and cultured on the nanofilms for up to 21 days. Focal adhesion size, cell proliferation, cell alignment, ALP levels, calcium and phosphate deposition, and Ca∶P ratios were assessed at various time points to investigate the role of PMMA nanotopography on osteogenic differentiation in MSCs.

## Materials and Methods

### Manufacturing of PMMA films

**Figure 1:**
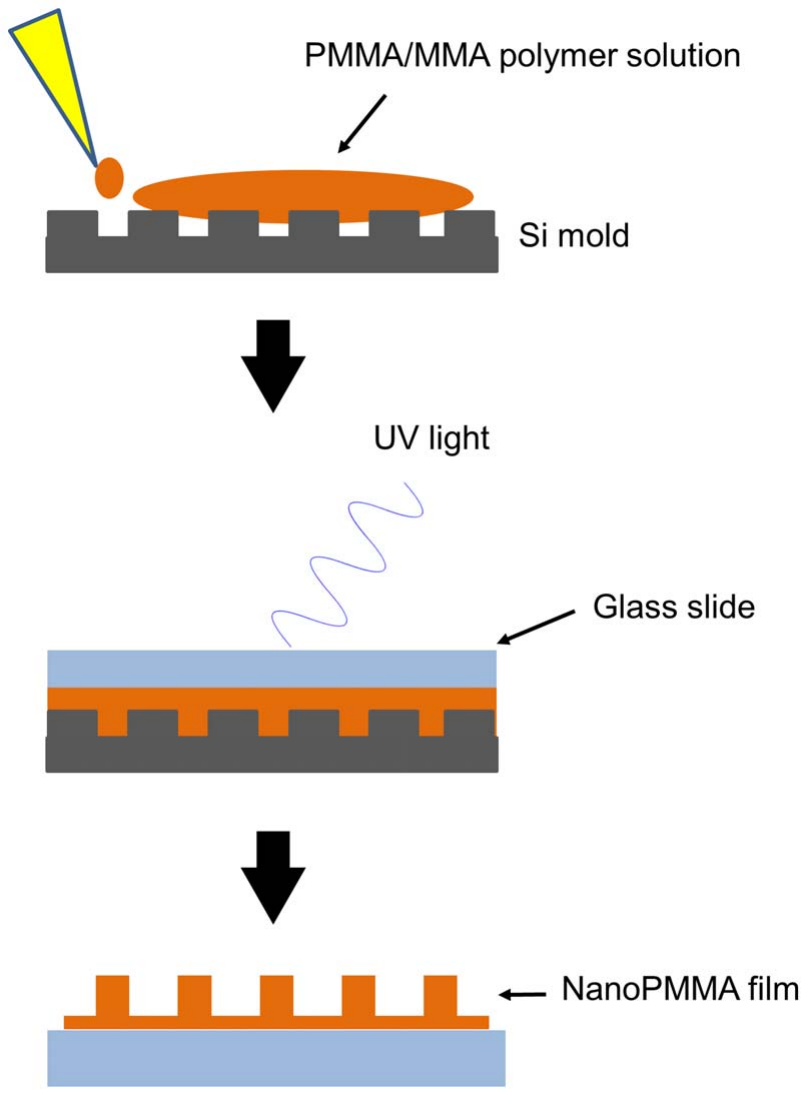
Capillary assisted UV lithography polymerization method was used to produce PMMA films with nanotopography. A precursor solution of PMMA and MMA was placed on a Si mold containing nanoscale gratings. A glass slide was placed on top of the precursor solution, which was then exposed to UV light for one hour. After polymerization the mold was removed. The final product was a PMMA film with nanoscale gratings of varied width and depth. See Table 1 for dimensions.

Films were made using a precursor solution of (poly) methyl methacrylate (PMMA) (M_w_: 120,000 g/mol) dissolved in methyl methacrylate (MMA) (8% wt./wt.) (all chemicals are from Sigma, Saint Louis, MO, unless otherwise specified). A photoinitiatior, 2,2-dimethoxy-2-phenylacetophenone (DMPA) (Acros Organics) (2% wt/wt), was added to the solution prior to polymerization. The PMMA precursor solution was deposited on (heptadecafluoro-1,2,2,2-tetrahydrodecyl) trichlorosilane (FTDS) (Gelest, Morrisville, PA) coated silicon molds (LightSmyth Technologies, Eugene OR). The silicon (Si) molds were patterned with square wave gratings of different depths and widths in order to create PMMA films with varied nanotopography (Table 1). No. 1 coverglass slides (Fisher Scientific, Pittsburgh, PA) were coated with ∼1 mM (3-acryloxy propyl) methyl dichlorosilane (APMDS) (Gelest) under vacuum overnight in a solution of dimethyl formamide (Fisher Scientific) and 1, 4- benzoquinone (9.25 mM). APMDS coated slides were rinsed in n-heptane and then dried with nitrogen gas. The pre-cursor solution was placed on top of the silicon molds and coated glass slides were placed on-top of the pre-cursor solution and subjected to UV-light ∼365 nm (3.1 mW/cm^2^) for an hour (Figure 1). Smooth PMMA films absent of topography were also manufactured. PMMA films will be designated by the following names for simplicity and clarity: smooth, G415, G303, and G140.

**Table 1:** Nomenclature for PMMA films with nanotopographic features.

Name	Period (nm)	Groove depth (nm)	Duty cycle (%)	Line width (nm)
G415	833	200	50	416
G303	606	190	50	303
G140	278	110	50	139

### Characterization of PMMA films with atomic force microscopy

To assess the topographic surface features of nanoPMMA films, a Dimension Icon scanning probe microscope (Bruker, Camarillo, CA) was used for imaging. Specifically, NCH-10 silicon probes (NanoAndMore USA, Lady's Island, SC) were used to scan the PMMA substrates.

### Determination of contact angles

A Ramé-Hart standard contact angle goniometer (model 200-F1; Succusunna, NJ) with Drop Image Advanced software was used to measure contact angles on PMMA films. Data was acquired as volume was added or retracted from the surface to determine the advancing and receding contact angles. A minimum of three independent measurements were made on each surface using deionized water and dioodomethane (DIM).

### Surface free energy calculations

We used equations first presented by Owens and Wendt [26] and used recently for nanoPMMA films to determine surface free energy.[27] Briefly, the relation between contact angle and surface free energy (SFE) is: 
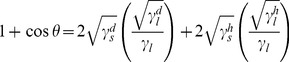
(1)where γ*_l_* = γ*_l_^d^*+γ*_l_^h^* and γ*_s_* = γ*_s_^d^*+γ*_s_^h^* are the surface free energies of a given liquid and solid. The contributions from different intermolecular forces are denoted by the superscripts: h and d refer to the hydrogen bonding and dispersion force components, respectively. Thus, using contact angles from two different liquids (water and DIM; see above), two equations can be solved simultaneously for γ*_s_^d^* and γ*_s_^h^*, to obtain γ*_s_*, in this case the SFE of PMMA. We used the average advancing angles to compute a SFE value. Additionally, we used γ*_l_^h^* = 49.5 mN/m, γ*_l_^d^* = 1.3 mN/m and γ*_l_^h^* = 51 mN/m, γ*_l_^d^* = 21.8 mN/m for the respective DIM and water surface free energy components.

### Cell culture

Human bone marrow-derived MSCs were obtained from a commercial source (Lonza, Walkersville, MD) at passage 2. As part of the manufacturer's quality control, MSCs were tested for purity by flow cytometry and for their ability to differentiate into osteogenic, chondrogenic and adipogenic lineages and are positive for the cell surface markers CD105, CD166, CD29 (integrin β1), and CD44, and negative for CD14, CD34 and CD45. MSCs were maintained in high glucose (4.5 g/L) Dulbecco's modified Eagle medium (DMEM, Invitrogen, Carlsbad, CA) supplemented with 10% fetal bovine serum (FBS, Invitrogen). All cultures were incubated at 37°C and 5% CO_2_. Media were changed every 2–3 days. MSCs were routinely expanded in 2D cultures and harvested with 0.05% Trypsin-EDTA (Invitrogen). Cells were used between passage 6 and 10.

### Initial cell adhesion and proliferation

PMMA substrates were cut to approximately 1 cm×1 cm and sterilized under UV light for ten minutes. After UV sterilization, the substrates were rinsed twice with sterile Dulbecco's phosphate buffered saline (PBS) (Invitrogen) pH 7.4, and then placed in 24 well plates prior to cell seeding. To determine if nanoPMMA influenced the number of adherent MSCs, cells were seeded at 5000 cells/cm^2^ and allowed to adhere for four hours in osteogenic growth media (OGM), consisting of alpha-minimum essential media (αMEM, Invitrogen), 20% FBS, 2 mM L-glutamine (CellGro, Manassas, VA), 1% penticillin/streptomycin (CellGro), and 5 mg/mL gentamicin (Invitrogen). After 4 hours, cells were rinsed with PBS, and then fixed with 4% paraformaldehyde in PBS, stained and imaged as described below. At least 5 different images (10X images) were observed on three separate surfaces. Values were normalized to the average number of cells observed on smooth PMMA surfaces. To determine the influence of nanoPMMA on proliferation, MSCs were seeded at an initial density of 2000 cells/cm^2^ and allowed to proliferate in OGM. At day 4, 7, and 14, cells were rinsed with PBS, and then fixed with 4% paraformaldehyde in PBS. Cells were then rinsed with PBS three times. Cells were stained with 4′,6-diamidino-2-phenylindole dihydrochloride (DAPI) in PBS (1∶5000) for 10 minutes to identify their nuclei. After staining, MSCs were rinsed with PBS and then imaged on an Olympus IX81 microscope equipped with a 100 W high-pressure mercury burner (Olympus America, Center Valley, PA), a Hamamatsu Orca II CCD camera (Hamamatsu Photonoics, K.K., Hamamatsu City, Japan), and Metamorph Premier software (Molecular Devices, Sunnyvale, CA). Cells that stained positive for DAPI were manually counted using Image J (NIH, Bethesda, MD). At least six (10X) images were analyzed for each condition. The number of cells per field was converted to cells per area, and then normalized to the initial number of cells seeded (2000 cells/cm^2^). Three independent measurements were made and averaged.

### Visualization of actin and vinculin using fluorescence microscopy

Cells were seeded at 2000 cells/cm^2^ on PMMA films (sterilized as previously described) and grown in osteogenic base media (OBM), consisting of OGM, 10 mM β-glycerol phosphate, and 50 µg/mL L-ascorbic acid (Fisher Scientific). At day 1, 4, and 7, cells were washed with PBS, permeabilized with 10 mM N-2-hydroxyethylpiperazine-N′-2-ethanesulfonic acid (HEPES) pH 6.9, 50 mM NaCl, 3 mM MgCl_2_, 300 mM sucrose, and 1 mM ethylene glycol tetraacetic acid (EGTA) with 0.5% Triton X-100 for one minute, washed, and then permeabilized again for 30 seconds in the same buffer before fixation. Cells were then fixed with 4% paraformaldehyde in PBS. These short permeabilization steps prior to fixation remove cytosolic vinculin as previously described [28]. Cells were washed with Tris-buffered saline (250 mM Tris, 27 mM KCl, 1.37 M NaCl pH 7.4; TBS, Fisher Scientific)+0.1% Triton X-100 (TBS-T) prior to blocking. Cells were then blocked with Abdil (2% bovine serum albumin in TBS-T) for 20 minutes and washed again with TBS-T. Cells were then incubated with Oregon Green 488 Phalloidin (Invitrogen) to stain F-actin (1∶40) and mouse anti-human vinculin (1∶250) to label focal adhesions in Abdil for 45 minutes. Following incubation, a TBS-T wash was used to remove unbound antibodies. Cells were then incubated for 45 minute with goat anti-mouse Alexa Fluor 594 (Invitrogen) in Abdil (1∶450). Cells were then washed in TBS-T at least three times. Stained MSCs on PMMA substrates were imaged on the Olympus IX81 microscope equipped as described above.

### Measurement of Cell Alignment

To determine the alignment of MSCs with respect to their underlying topography measurements were made using Image J at day 1, 4 and 7. First, the angle of the gratings was determined relative to the horizontal using the measure angle feature in Image J. Next, the long axis (with respect to the horizontal) of a minimum of 36 cells on each substrate was measured. The orientation angle was then computed. An angle of 0° represents perfect alignment with the underlying topography, 45° represents random orientation, and 90° represents a cell that is perpendicular to the topography.

### Focal Adhesion size

Images were taken on the Olympus IX81 as described above, converted to grey scale and processed with a high-band pass filter using Metamorph Premier (v 7.7.2.0, Molecular Devices, Sunnyvale, CA) to sharpen and enhance the focal adhesions. Focal adhesion size was determined by tracing a line along the length of the adhesion of interest and measured using the ‘Measure’ feature in Image J similar to the procedure described previously [29]. A minimum of 90 adhesions were analyzed per condition.

### Osteogenic differentiation

PMMA films were prepared and sterilized as described above. For osteogenic differentiation, MSCs were seeded at 5,000 cells/cm^2^ in a 24-well plate for functional assays. Cells were cultured in OBM as described above and as previously reported [30], [31].

### Alkaline phosphatase assays

Cellular alkaline phosphatase (ALP) activity was measured at 1, 7, and 14 days, as previously described [12]. Briefly, cells were rinsed with PBS and lysed using passive lysis buffer (Promega, Madision, WI). 10 mM Tris-HCl (pH 7.4) was added to the lysates; they were then briefly vortexed and centrifuged at 10,000 rpm for 10 minutes. ALP activity was assayed at 37°C in a buffer containing 100 mM glycine (Biorad, Hercules, CA) and 1 mM MgCl_2_ (Fisher Scientific) (pH 10.5) for 20 minutes using *p*-nitrophenol phosphate (pNPP; Fisher Scientific) (50 mM) as a substrate. The reaction was terminated using 0.1 N NaOH (Fisher Scientific). The amount of pNPP liberated was determined spectrophotometrically using a Genova MK3 (Jenway, Staffordshire, United Kingdom) spectrophotometer at 405 nm. ALP activity (units/mL) was normalized by total protein levels for each specimen. A minimum of three samples were assayed for each condition.

Cells were also stained to visualize ALP activity. After an initial PBS rinse, cells were fixed with 4% paraformaldehyde in PBS for one minute and rinsed with ultrapure water twice. Cells were then rinsed in TBS-T and stained with a 0.08 M Tris buffer (pH 8.2) with 0.8 mg/mL of Fast Blue RR salt and 67.2 µg/mL napthol AS-MX phosphate powder for 10 minutes while protected from light, similar to procedures described elsewhere [17], [32]. After staining, cells were rinsed in DD water for one minute and then rinsed with TBS-T prior to imaging. Images were taken on an Olympus IX81 with a DP25 color camera.

### Von Kossa staining

Cells were rinsed 2X in PBS and then fixed in 4% paraformaldehyde in PBS at 4°C for 30 minutes after 14 and 21 days in OM. After fixation, cells were rinsed in ultrapure water 3X and then immersed in 5% AgNO_3_ and subjected to UV light (∼365 nm) for 40 minutes. After UV exposure cells were rinsed 3X in DD water. The cells were then rinsed in sodium thiosulfate for 3 minutes and rinsed in DD water 3X. Images were taken on an Olympus IX81 with a DP25 color camera.

### Calcium quantification

Calcium content in osteogenic cultures was quantified using the ortho-cresolphthalein complexone (OCPC) method, as previously described [33], [34]. Cells were initially seeded at 5000 cells/cm^2^ on sterile PMMA films as described above. To account for any differences in cell number, MSCs were counted by quantifying DAPI-stained nuclei as described above; the total numbers of cells after 14 and 21 days of culture on each substrate were used to normalize the total calcium levels. After cell number determination, cells were washed in PBS before incubation in 1 mL of 1 N acetic acid overnight. The OCPC solution was prepared by adding OCPC to DD water with 1 N KOH (Acros) and 1 N acetic acid (Fisher Scientific). The dissolved solutions (10 µL per replicate) were then mixed with a working solution (300 µL per replicate) of OCPC solution and ethanolamine/boric acid/8-hydroxyquinoline buffer. Absorbance values were recorded using a Thermo Scientific Multiskan Spectrum spectrophotometer at 570 nm. Calcium values were quantified via a standard curve from 0 to 150 µg/mL. Specimens and standards were assayed in triplicate. Three samples of each condition were analyzed. Values were normalized to account for potential differences in cell number and substrate surface area, and then by the average calcium level observed after 14 days on smooth PMMA.

### Scanning electron microscopy (SEM)

At day 21, cells were washed in PBS and subsequently fixed for 20 minutes in 4% PFA in PBS. After fixation, cells were washed twice in ddH2O and allowed to air dry overnight. The specimens were sputter-coated with gold and examined with a Phillips XL30FEG SEM equipped with an EDAX Phoenix X-ray energy dispersive spectrometer (XEDS). A working distance of 10 mm and an accelerating voltage of 15 kV were used for XEDS mineral chemical micro-analysis. A minimum of three areas from three distinct fields of view were scanned for 60 seconds to determine Ca∶P ratios. Hydroxyapatite (HA) and mouse femur were used as controls. Briefly, hydroxyapatite powder was embedded in a viscous polymer resin and allowed to dry overnight at 40°C. The femur section was harvested from an 8-week old mouse and fixed in 4% paraformaldehyde in PBS for 2 days. The tissue was dehydrated in graded solutions of ethanol over 5 days, and then embedded in methyl methacrylate (MMA) using benzoyl peroxide, nonylphenyl polyethylene glycol acetate, and N,N-dimethyl-p-toluidine. After embedding, the tissue was sectioned into ∼100 µm thick slices. Both HA and femur controls were sputter-coated with gold.

### Statistical analysis

Statistical analyses were carried out using GraphPad Prism software. All data were assessed for normality (where appropriate) using the D'Agostino-Pearson normality test. When appropriate, one-way ANOVA or the Kruskal-Wallis analysis with the respective post-hoc (Tukey or Dunn's) test was performed. Data are reported as means ± standard deviations. Significance was set at *p*<0.05.

## Results

### Nanotopography on PMMA films is confirmed by AFM

**Figure 2:**
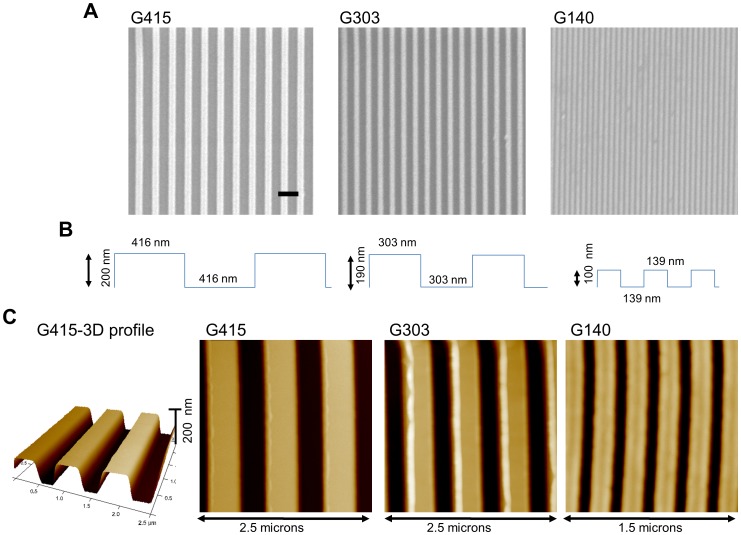
Capillary assisted UV lithography produced thin film substrates with nanotopography. (A) Scanning electron microscopy (SEM) was utilized to image nanoPMMA films. Micrographs confirm the presence of nanoscale dimensions in all three of the different film types utilized in this study: G415, G303, and G140 (scale bar  = 1 micron). (B) Illustrations show the approximate dimensions (height and width) of the PMMA films. (C) Atomic force microscopy was used to confirm that PMMA films had the expected nanotopographic features (see Table 1 for dimensions).

PMMA films with nanotopographic features were fabricated using UV-assisted capillary force lithography (Figure 1). The topographic dimensions of the films were confirmed using AFM. As expected, the films had similar dimensions to the Si master molds that were used for polymerization, and the substrates were designated G415, G303, and G140 based on these dimensions (Table 1; Figure 2).

### Contact angle measurements illustrate anistropic wettability

**Figure 3:**
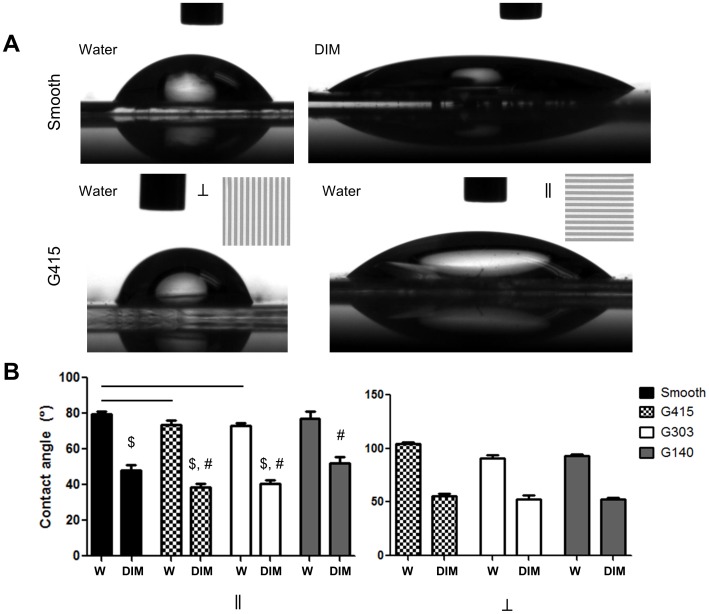
Contact angle measurements show anisotropy dependent on surface orientation. (A) Images illustrating wettability of water or diiodomethane on smooth or G415 PMMA films. Insets on G415 images show the direction of the underlying topography (⊥ indicates a drop perpendicular to the long axis of the topography; ∥ indicates a drop parallel to the long axis of the topography). Contact angle anisotropy can be observed on the G415 surfaces and also occurred on other nanoPMMA films (data not shown). (B) Graphs illustrate the quantification of the contact angles both parallel and perpendicular to the grating direction. Significant differences were seen between smooth PMMA and G415 and G303 (parallel) for water and DIM ($) contact angles ($). All surfaces, when perpendicular contact angles for nanoPMMA were compared to smooth PMMA for both liquids, were significant. W indicates contact angle measurement of water; DIM indicates indicates contact angle measurement of diiodomethane. All significance was p<0.05 (or smaller), n≥3.

Measurements of contact angle using water and diiodomethane revealed contact angle anisotropy (Figure 3). Our results are in agreement with reports that suggested that as groove depth increases the contact angle decreases [35] and anistropic wetting increases as groove depth increases [36] as observed in our contact angle measurements on nanoPMMA parallel to the gratings (Figure 3B). Additionally, measurements of contact angles orthogonal to the gratings showed an increase. This increase is due to pinning at the grating wall as additional energy is needed for the drop to continue spreading orthogonal to the gratings. Thus, spreading is preferential along the grooves rather than perpendicular to the grooves as the energy cost is lower. [37]

### Surface free energy calculations illustrate slight differences and anisotropy

Using the contact angle measurements, we then calculated the surface free energy of each surface using the approximation from Owens and Wendt for polymers. [26] We realize that this approximation may not fully model the physical situation, however, to the best of our knowledge, most theoretical thermodynamic approximations for microscale and nanoscale gratings have yet to be verified experimentally.[38]–[40] Experimental validation is important as the Wenzel and Cassie theories do not always hold up in practice; three phase contact line approaches seem to be more valid. [41] One report validated contact angle measurements parallel to micro and nanogratings (in agreement with Wenzel) but did not discuss SFE estimations different than Owens and Wendt. [42] The surface free energy of smooth PMMA was calculated to be 40.0 mN/m, very similar to the 40.2 mN/m value reported by Owens and Wendt. Our SFE calculations for nanoPMMA revealed changes in values depending on the direction of measurement (Table 2). SFE parallel to the gratings was higher for G415 and G303 surfaces and slightly lower but similar for G140 surfaces compared to smooth PMMA SFE. Perpendicular SFE values were slightly lower than smooth PMMA SFE and lower than SFE for nanofilms parallel to nanogratings. These values are similar to those reported previously. [27] Our calculations confirm nanoPMMA film anisotropy.

**Table 2:** Calculated surface free energy values for PMMA films.

	(mN/m)	Smooth	G415 ∥	G303 ∥	G140 ∥	G415 ⊥	G303 ⊥	G140 ⊥
	γ*_s_^d^*	35.4	40.2	39.4	33.1	31.0	32.6	32.8
	γ*_s_^h^*	4.6	5.7	6.0	6.1	0.1	1.6	1.2
Total	γ*_s_*_, *PMMA*_	40.0	45.9	45.5	39.2	31.1	34.2	34.0

### MSCs cultured on nanotopography show altered alignment and proliferation

**Figure 4:**
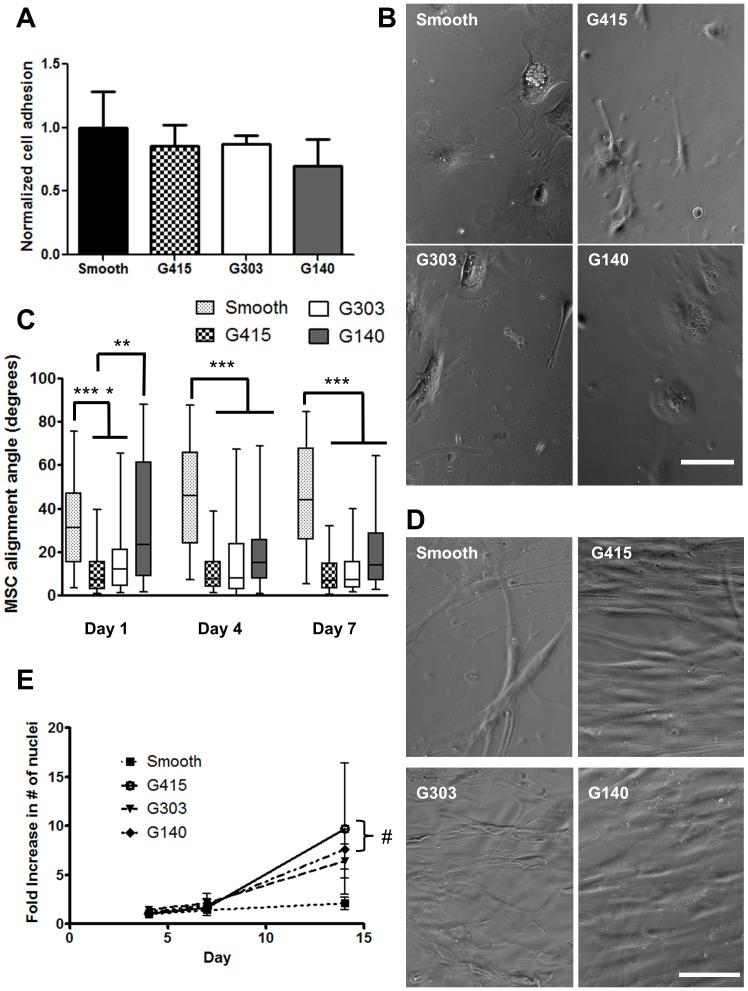
Nanotopography modulated MSC alignment and proliferation but did not modulate initial adhesion. (A) Similar numbers of MSCs adhered to all PMMA surfaces after four hours as quantified by DAPI stained cells. (B) Phase contrast images illustrate that cell adhesion was similar on all PMMA surfaces after four hours. Scale bar represents 100 µm. (C) Quantification of MSC alignment revealed significantly more MSC alignment (denoted by alignment angles closer to 0°, parallel to the underlying topography) on G415 and G303 substrates relative to smooth PMMA at all times points. Significant differences in alignment were also observed on G140 substrates (relative to smooth PMMA) at day 4 and 7. Data are plotted in a box and whiskers format, with the horizontal bars representing the medians, the boxes denoting the 25th and 75th confidence intervals of the data, and the whiskers denoting the boundaries of the 5th and 95th intervals. A minimum of 36 cells were analyzed for each of the conditions at each time point (nearly all the data sets were non-normally distributed, hence Kruskal-Wallis analysis was used, see Materials and Methods). (D) MSCs cultured on patterned PMMA aligned parallel to the direction of the nanotopography, as shown in these representative images following 14 days of culture. Scale bar represents 100 µm. (E) MSCs grown on G415 (p<0.005) and G140 (p<0.05) substrates proliferated at statistically greater rates compared to smooth PMMA after 14 days. Error bars represent standard deviation, n = 3 for day 4, 7 and n = 5 for day 14 (* *p*<0.05, ** *p<0.01*, *** p<0.005).

Initial cell attachment was not significantly altered on nanotopography (Figure 4A). Cells did start to elongate after 4 hours on nanotopography (Figure 4B). MSCs preferentially oriented parallel to the alignment of the nanotopography (Figure 4C,D). At day 1, cells cultured on G415 and G303 surfaces were significantly more aligned compared to those cultured on smooth and G140 PMMA. On G415, G303, and G140 PMMA films, MSCs had an elongated shape and were predominately aligned parallel to the underlying topography (Figure 4D). Cells grown on smooth PMMA were spread but showed no preferential alignment (Figure 4D). Quantitatively, nanotopographic substrates of all feature sizes significantly influenced cell alignment on both day 4 and day 7 compared to smooth PMMA controls (Figure 4C). Cell proliferation was assessed by counting DAPI-stained cell nuclei on days 4, 7, and 14. Increases in cell nuclei were not significantly altered on PMMA nanotopography compared to smooth controls at day 4 and day 7 (Figure 4E). By day 14, total cell number was significantly higher on all topographies compared to smooth controls. These data suggest that topography can be influential on cell proliferation after two weeks but does not significantly impact proliferation at earlier time points (day 4 and 7).

### MSCs cultured on nanotopography exhibit altered cytoskeletal structures and focal adhesions

**Figure 5:**
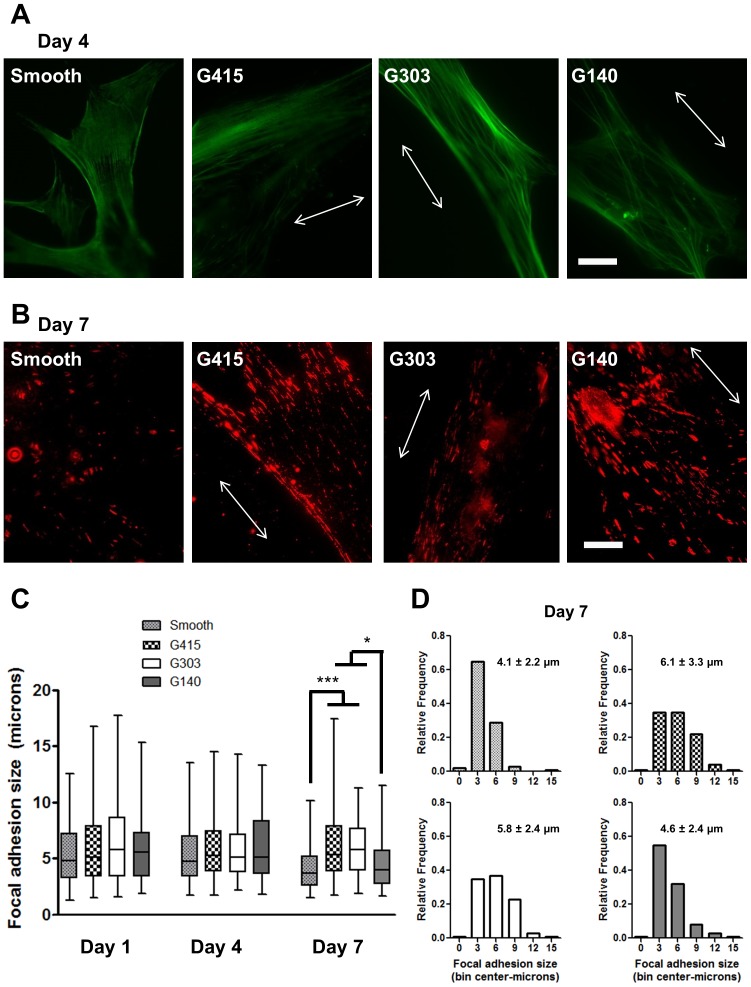
MSCs grown on nanotopography exhibit aligned actin cytoskeletons and more elongated focal adhesions. (A) Representative fluorescent photomicrographs of MSCs cultured on PMMA substrates for 4 days revealed the aligned orientation of the actin cytoskeleton in cells cultured on patterned substrates. Arrows indicate the orientation of the underlying topography (in A and B). Scale bar represents 25 µm. (B) Representative fluorescent photomicrographs (contrast enhanced to show differences) of MSCs cultured on PMMA substrates for 7 days following permeabilization, fixation, and staining of vinculin revealed qualitatively more elongated focal adhesions on substrates with larger topographic features (G415). Scale bar represents 25 µm. (C) Quantification of (unenhanced images of) focal adhesion size using Image J from cells cultured for 1, 4, and 7 days on PMMA substrates revealed no significant increases in focal adhesion size on the nanoPMMA substrates relative to smooth controls on Day 1 and Day 4. On Day 7, focal adhesions were significantly longer on G415 and G303 films compared to G140 and smooth PMMA surfaces. Data are plotted in a box and whiskers format, with the horizontal bars representing the medians, the boxes denoting the 25th and 75th confidence intervals of the data, and the whiskers denoting the boundaries of the 5th and 95th intervals. A minimum of 90 adhesions was assessed per condition (nearly all the data sets were non-normally distributed, hence Kruskal-Wallis analysis was used, see Materials and Methods, * *p*<0.05, *** p<0.005). (D) Histograms of the focal adhesion size distributions at day 7 showed that larger adhesions are present more frequently in cells cultured on the G415 and G303 films. The numbers on each graph represent the mean (+/−S.D.) adhesion size (in µm) for MSCs cultured on the PMMA substrates.

F-actin in cells grown on nanotopography was organized parallel to the underlying surface topography, while cells cultured on smooth surfaces exhibited F-actin arranged randomly (Figure 5A). Vinculin, a key structural component of focal adhesions [43], [44], also showed altered expression patterns on substrates containing nanotopography. Vinculin staining from day 7 cultures revealed elongated focal adhesions that were aligned parallel to the underlying topography (Figure 5B). Focal adhesions in cells grown on smooth films were randomly arranged around the cell periphery and were not as elongated. Quantification of focal adhesion size using Image J revealed that they were not significantly elongated at day 1 and day 4 on G415, G303, and G140 films compared to smooth PMMA (Figure 5C). After 7 days, focal adhesions were significantly longer on G415 and G303 films compared to G140 and smooth PMMA surfaces (Figure 5B,C). Histograms of the focal adhesion size distributions at day 7 not only revealed the differences in focal adhesion lengths between smooth PMMA and nanotopographic films, but also showed that the larger adhesions are present more frequently in cells cultured on the G415 and G303 films (Figure 5D).

### Nanotopography did not enhance ALP activity in MSCs

**Figure 6:**
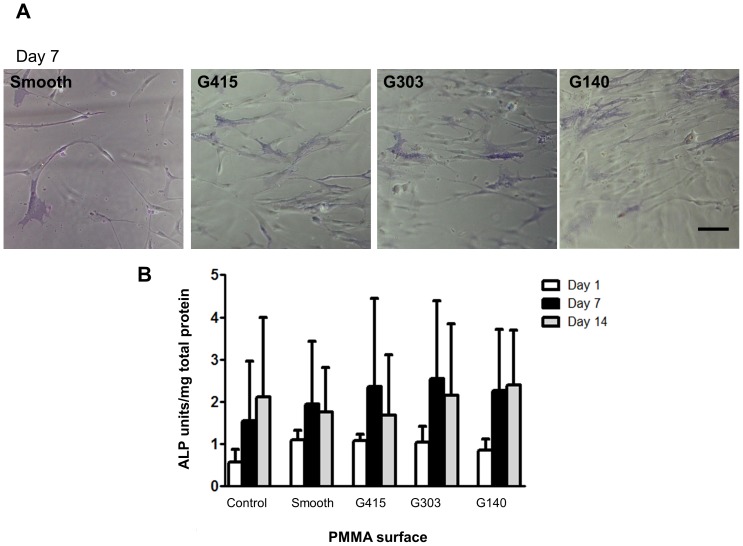
Alkaline phosphatase activities were not altered by culturing MSCs on nanotopography. (A) Micrographs of MSCs stained for active ALP after 7 days in osteogenic media (OBM) revealed no qualitative differences on PMMA nanotopgraphy. Scale bar represents 100 µm. (B) Quantification also revealed no significant differences in ALP activity for MSCs grown on nanotopography versus smooth PMMA substrates. The control group represents MSCs grown on smooth PMMA in osteogenic growth media (OGM).Data represent mean +/− standard deviation (n = 3 for day 1 and n = 5 for day 7, 14).

ALP, a marker of the early stages of osteogenic differentiation [45], was assessed using the Fast Blue RR stain at day 1, 7, and 14, (Figure 6A), illustrating that ALP activities of the MSCs was similar on all substrates on day 7. To verify these qualitative observations, ALP activities were also quantified using the colorimetric pNpp assay. Results from this assay revealed that ALP activities increased with culture time on all substrates, but confirmed that nanotopography induced no significant changes (relative to smooth controls) after 1, 7, or 14 days (Figure 6B).

### Calcium levels are minimally enhanced on nanotopography at day 14

**Figure 7:**
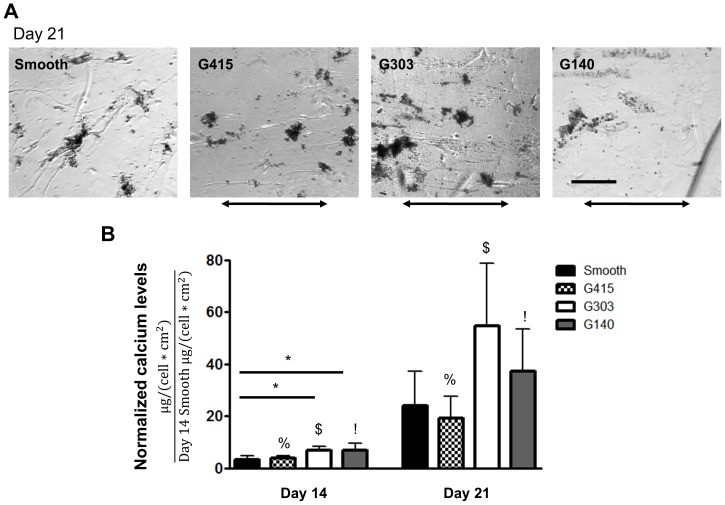
Calcium deposition was minimally influenced on nanotopgraphy. (A) Representative phase microscopy images illustrating mineral formed on PMMA films after MSCs were grown for 21 days in osteogenic mineralization media (OBM). Arrows indicate the direction of the underlying topography. Scale represents 200 µm. (B) Quantification of total calcium levels showed that G303 and G140 PMMA nanotopographic substrates supported slightly higher levels of calcium deposition on day 14 (* *p*<0.05). However, there were no significant differences after 21 days (n = 3). Symbols %, $ and ! all indicate significant differences (* *p*<0.05) between day 14 and day 21 time points for the respective conditions.

To assess the influence of nanotopography on the ability of MSCs to produce a mineralized matrix, the amount of deposited calcium was quantified after 14 and 21 days of culture using the OCPC assay (Figure 7A, B). To ensure that differences in cell proliferation on the PMMA films had no impact on the levels of calcium observed, the results were normalized by cell number (see Materials and Methods for details). Increases in calcium deposition on nanotopography (G303 and G140 films) were observed relative to smooth PMMA after 14 days (Figure 7B). No significant differences were observed after 21 days in culture when comparing nanotopography to smooth PMMA, though calcium levels were approximately 2 to 3 times higher on G303 and G140 surfaces. Significant increases in calcium deposition occurred when comparing G415 at day 21 versus day 14 (with similar results for G303 and G140 films); a statistically insignificant increase was observed on smooth PMMA.

### Mineral Ca∶P ratios are influenced by nanotopography

**Figure 8:**
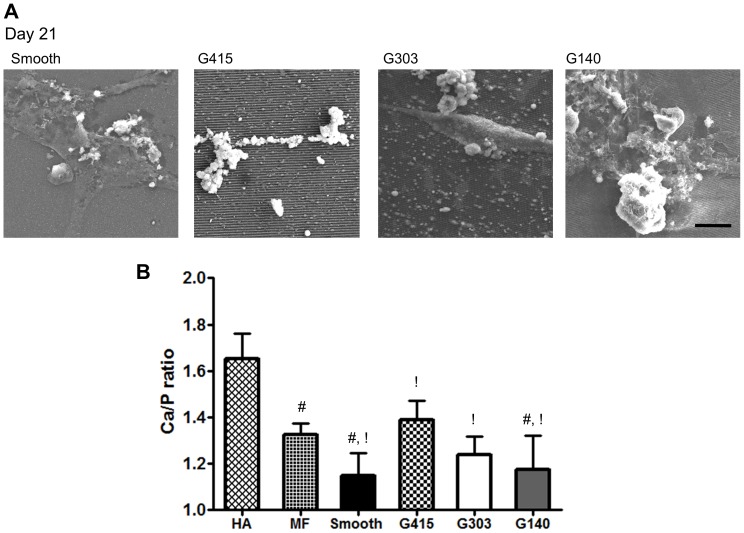
Ca∶P ratios of mineral deposited on G415 substrates were similar to those found in mouse bone. (A) SEM micrographs revealed the presence of mineral after MSCs were cultured for 21 days in osteogenic media (OBM) on both smooth and nanotopographic PMMA films. Alignment of mineral was observed on the G415 substrate. Scale represents 10 µm. (B) Quantification of Ca∶P ratios via XEDS revealed no significant differences between the G303, G140, and smooth PMMA substrates. The G415 films supported mineral with significantly higher Ca∶P ratios compared to the other PMMA films, with values similar to those of native bone from mouse femur (MF). The Ca/P ratio of purified hydroxyapatite (HA) was quantified as a positive control. Symbols # and ! indicate that Ca∶P ratios observed in MF and on G415 substrates were significantly higher (*p*<0.05, some comparisons showed greater significance) than other PMMA surfaces that share the same symbol, n≥6.

To assess composition, mineral deposits were stained via the Von Kossa method to visualize the presence of phosphates [46]. Topographic and smooth surfaces stained positive for phosphates (data not shown). Additionally, deposited mineral was observed using SEM (Figure 8A). To determine the presence of calcium and phosphorous, XEDS was used. Analysis showed that there were significant differences in the Ca∶P ratio (Figure 8B). Mineral deposited on G415 films was the most similar to bone (Ca∶P ratio 1.39 and 1.32 respectively) For reference, we observed the Ca∶P ratio in hydroxyapatite to be 1.65 (theoretical ratio 1.67). Ca∶P ratios in mouse femur were significantly higher than on G140, and smooth PMMA films and mineral on G415 films Ca∶P ratios were significantly higher than on G303, G140, and smooth PMMA films.

## Discussion

The ECM is a multifaceted instructive material that plays an important role in both normal and pathologic development, and is central in many regenerative strategies. Over the past 10–15 years, an increasing number of studies emphasizing the ECM's physical features, including its mechanical rigidity and its topography, have shown that cells sense and respond to ECM cues beyond just the adhesive ligands known to bind integrin receptors. In this study, we created PMMA substrates with nanoscale gratings via capillary assisted UV lithography and used them to assess the potential functional influence of ECM topography on the osteogenic phenotype of MSCs. Cell alignment and proliferation were both influenced by topography, consistent with numerous prior studies. However, assessments of calcium quantity and composition showed that PMMA nanotopography, at least of the feature sizes studied here, was not in fact a strong driver of an osteogenic phenotype *in vitro*.

After quantitatively confirming the features molded on PMMA films, we investigated the wettability and calculated surface free energies from contact angle measurements. The anisotropy of the surface features was reflected in the contact angle measurements and the surface free energy calculations. Our calculated surface energies for smooth PMMA were similar to values reported by Owens and Wendt and our nanoPMMA SFE values were similar to those in a previous report [26], [27]. Next, we investigated the influence of nanotopography on the shape, alignment, proliferation, and initial adhesion of MSCs on PMMA gratings. Our results confirm that topography did alter cell shape, alignment at day 1, 4, and 7, and proliferation at day 14, but did not influence the number of MSCs that initially attached to the surface. Numerous other studies have reported that nanoscale gratings induce similar changes in the alignment of a wide range of cell types, including smooth muscle cells [47], fibroblasts [48], rat osteoblasts [49], cardiac myocytes [50], and even MSCs [3], [51], [52]. Prior studies have suggested that cell orientation (of macrophages and fibroblasts) may also be dependent on groove depth [53], [54]. Our results showed greater alignment on nanopatterns with greater depth (G415-depth 200 nm; see Table 1); however, in our study, grating width also varied. Thus, the increased alignment we observed cannot solely be attributed to increased grating depth. Furthermore, nanoscale gratings on PMMA films increase the available surface area (up to 79% greater surface area) compared to smooth PMMA films. This increase in surface area combined with the increased cell alignment may increase the available space for cell growth, providing a possible explanation for the increased proliferation observed on PMMA substrates with nanotopography.

Because we observed changes in shape, alignment, and (to a lesser degree) proliferation of MSCs cultured on nanotopography, we next examined the actin cytoskeleton and focal adhesions. Fluorescent images of the actin network revealed that the cytoskeleton was oriented parallel to the underlying topography when MSCs were cultured on patterned substrates. The focal adhesions also exhibit some degree of aligned orientation parallel to the topography, but the effect was less pronounced on the G140 substrates (those with the smallest gratings, and closest in topography to the smooth substrates). Quantification of the focal adhesion sizes showed that the G415 and G303 substrates not only supported more elongated, fibrillar adhesions than the G140 or smooth substrates, but also that these larger adhesions were present in MSCs at higher frequencies. A prior study also investigated focal adhesion size of human osteoprogenitor cells grown on PMMA surfaces with nanopits or microgratings, and reported a decrease in focal adhesion number and size on topographic surfaces compared to planar PMMA [10]. Similar findings were reported on polycarbonate [55]. Another study reported a decrease in the levels of zyxin, a molecular marker of mature focal adhesions, in hMSCs grown on 350 nm gratings of polydimethylsiloxane, suggesting that topography may disrupt focal adhesion maturation [4]. In our case, certain topographic sizes (G415 and G303) actually supported larger adhesions than smooth substrates, suggesting that the correlation between adhesion size and nanotopography is likely a strong function of the size, shape, and chemistry of the nanoscale feature.

To characterize the influence of PMMA nanotopography on the osteogenic phenotype of MSCs, we focused on ALP activity and mineral deposition. ALP effectively increases the relative concentration of extracellular phosphate leading to more favorable conditions for hydroxyapatite formation [22]. Our results showed that MSCs expressed ALP to similar levels and activities on all PMMA substrates investigated, regardless of topography. Characterization of mineral deposition via qualitative von Kossa (phosphate) staining and a quantitative calcium assay revealed no significant increase in mineralization on topography after 21 days of culture in the presence of osteoinductive supplements. However, our data do suggest that topography may alter mineral quality by modulating the Ca∶P ratio, as MSCs grown on G415 substrates produced mineral with a similar ratio as mouse bone. Other surfaces produced a highly calcium deficient mineral. *In vivo* bone is inherently calcium deficient, especially when newly formed, with reported calcium phosphate ratios of approximately 1.5 to 1.6 in rat and bovine specimens [56], [57]. Low *in vivo* Ca∶P ratios are often associated with osteogenesis imperfecta, which may be related to non-ideal collagen fibril sizes [58]. G415 surfaces may potentially accelerate the maturity of newly formed HA minerals due to a collagen matrix more representative of that found in bone. Additional investigation is needed to address this possibility.

Many prior studies have investigated the influence of substrate nanotopography on the osteogenic phenotype of MSCs, but there are substantial discrepancies that remain to be resolved. Several reports claim that nanoscale features can control the osteogenic differentiation of MSCs [14], [59], but the topography utilized in those studies was composed of nanoscale pits rather than the gratings used here. Likewise, numerous other studies have reported that nanoscale cues can induce the expression of osteogenic genes in MSCs [14], [59], [60], but did not characterize mineral quantity and quality as we did here. One study that characterized mineral deposited by rat osteoblast-like cells cultured on nanotopography showed that the mineral contained calcium and phosphorous and aligned parallel to the underlying topography [49]. However, neither the amount of calcium nor the Ca∶P ratio were assessed. Another study showed that rat MSCs grown on polystyrene nanogratings had lower ALP levels than did those grown on flat substrates, but there were no significant differences in calcium deposition on topography relative to flat controls [52]. Most similar to our study, hMSCs grown in osteogenic media on polyurethane gratings of 200 nm width and 300 nm depth showed increased deposition of calcium compared to smooth films at day 7, but not at day 14 or 21 [3]. Other sizes (700 nm and 2000 nm width) did not increase calcium deposition.

Besides differences in characterization methods and functional assessments of phenotype, there are many other possible reasons for the disparate observations regarding nanotopograhpy and MSC osteogenic differentiation in the literature. Amongst the most obvious are differences in material chemistry and topographic feature size. A wide variety of materials have been imprinted with topographic cues of a variety of shapes and sizes. In the case of nanotopographic gratings like those we have used here, there are studies that have used polystyrene [49], [52], polyurethane [3], and PDMS [20], [51], among others. Differences in material surfaces (due to different material chemistries, treatments, or topographies) are likely to influence the SFE [61] and thus the wettability [62], perhaps leading to changes in identity and conformation of adsorbed proteins [63]–[65]. Changes in protein conformation have been linked to differences in topography [64] and result in altered cell behavior [66]–[68]. Surface features that change the SFE landscape, therefore, may be at least partially responsible for the observed changes in cell behavior.

Our results suggest that the slight differences in calculated SFE had little influence on the osteogenic phenotype of MSCs. This finding indicates that either SFE differences observed here were not sufficient to alter the protein landscape or the competitive adsorption from FBS mitigated any changes in ECM identity, spatial presentation, or conformation that could have potentially enhanced the osteogenic potential of MSCs on nanoPMMA. Since ECM ligand identity [69], [70] and spatial presentation [71] are already known to be strong determinants of the osteogenic fate of MSCs, it may very well turn out that specific surface chemistries and nanotopographic features may indirectly alter ligand identity and spatial presentation to impact cell fate, perhaps in much the same way (and via the same mechanisms) as matrix elasticity. However, with the wide range of materials and topographies available for study, attaining consensus regarding the instructive role of topography on differentiation remains a significant challenge.

A number of previous studies combine physical topography with a single type of ECM protein (via coating, stamping etc.) [16], [17], [50], [66]. The single adhesion cue coupled with topography could be a strong driver of the various cell behaviors observed. In contrast, we investigated the influence of topography and uncontrolled protein adsorption from FBS on MSC osteogenic behavior in an attempt to replicate *in vivo* implant-protein environments [72]. Multiple ECM proteins in FBS are known to adsorb to surfaces, hence the osteogenic influence of one protein type may be masked by the presence of other proteins due to the abundance of multiple and varied adhesion epitopes. Disparate ligand identity has shown to play a varied role on MSC's osteogenic differentiation [70], [73], thus competition for a number of ECM adhesion epitopes likely hinders activation of an osteogenic phenotype, preventing any topographic differentiation enhancement from being evident.

In this study, we focused on PMMA because of its application as a bone cement in orthopedic applications [24], [25], motivated by the possibility that simply imprinting nanotopographic cues on an FDA-approved orthopedic material might enhance osteointegration and bone healing. Ultimately, whether or not nanotopography can be used to enhance bone formation and implant integration will depend on studies demonstrating its utility *in vivo*. A recent study created nanotopography on titanium implants. They found increased bone to implant contact area on most nanograting surfaces compared to the control at 4 and 8 weeks [1]. Mechanical stability of the implants was not assessed. Implants capable of promoting tissue integration and lamellar bone could reduce recovery time and reduce patient discomfort. Thus, topography may turn out to be important clinically, but further investigations are needed.

### Conclusion

In this study, we showed that MSC alignment, focal adhesion and cytoskeleton assembly, and proliferation are all influenced by nanotopographic gratings of PMMA in the 140–415 nm size range. However, using both qualitative and quantitative assessments of mineralization, we conclude that PMMA nanotopgraphy is a poor driver of the ostegenic differentiation of MSCs *in vitro*. Differences in Ca∶P ratios present in deposited mineral were influenced by topographic surfaces with specific feature sizes, suggesting that certain feature sizes might enhance maturation of deposited mineral.
